# Carboxin and Diuron Adsorption Mechanism on Sunflower Husks Biochar and Goethite in the Single/Mixed Pesticide Solutions

**DOI:** 10.3390/ma14102584

**Published:** 2021-05-16

**Authors:** Katarzyna Szewczuk-Karpisz, Agnieszka Tomczyk, Magdalena Celińska, Zofia Sokołowska, Marcin Kuśmierz

**Affiliations:** 1 Institute of Agrophysics, Polish Academy of Sciences, Doświadczalna 4, 20-290 Lublin, Poland; a.tomczyk@ipan.lublin.pl (A.T.); m.celinska@ipan.lublin.pl (M.C.); z.sokolowska@ipan.lublin.pl (Z.S.); 2 Analytical Laboratory, Institute of Chemical Sciences, Faculty of Chemistry, Maria Curie-Sklodowska University, Maria Curie-Sklodowska Sq. 3, 20-031 Lublin, Poland; marcin.kusmierz@poczta.umcs.lublin.pl

**Keywords:** simultaneous adsorption, pesticide, SEM, FTIR, XPS, Boehm titration, soil additives

## Abstract

The study focused on the adsorption mechanism of two selected pesticides: carboxin and diuron, on goethite and biochar, which were treated as potential compounds of mixed adsorbent. The authors also prepared a simple mixture of goethite and biochar and performed adsorption measurements on this material. The adsorbents were characterized by several methods, inter alia, nitrogen adsorption/desorption, Boehm titration, Fourier transform infrared spectroscopy and X-ray photoelectron spectroscopy. The adsorption study included kinetics and equilibrium measurements, in the solution containing one or two pesticides simultaneously. The adsorption data were fitted to selected theoretical models (e.g., Langmuir, Freudlich, Redlich–Peterson, pseudo first-order and pseudo second-order equations). Based on the obtained results, it was stated that, among all tested adsorbents, biochar had the highest adsorption capacity relative to both carboxin and diuron. It equaled 0.64 and 0.52 mg/g, respectively. Experimental data were best fitted to the pseudo second-order and Redlich–Peterson models. In the mixed systems, the adsorption levels observed on biochar, goethite and their mixture were higher for diuron and lower for carboxin, compared to those noted in the single solutions. The presented results may enable the development of new mixed adsorbent for remediation of soils polluted with pesticides.

## 1. Introduction

In recent years, environmental pollution by pesticides is becoming more and more serious [1]. Pesticides are chemicals widely used in agriculture, horticulture, forestry, veterinary medicine as well as for the impregnation of textiles and plastics. They are degradable substances, but most of them remain in the environment for very long time. They may be accumulated in all living organisms and involved in various trophic chains. Most of pesticides are designed as highly selective substances. However, in practice, they affect not only the pests but also other living organisms in the surrounding environment [2]. The toxicity of plant protection products (PPP) results from the presence of biologically active ingredients, emulsifiers, auxiliary substances and fillers [3]. Among the wide variety of pesticides, there are diuron and carboxin investigated in this paper.

Diuron (N-(3,4-dichlorophenyl)-N,N-dimethyl-urea) is a herbicide from the phenylamides group. It acts as a photosystem-II inhibitor. Diuron exhibits poor solubility in water and may contribute to disease development by inhalation, skin absorption and/or ingestion [4]. Maximum allowed level of this pesticide residue allowed in EU is: 0.01 mg/kg for fruits, vegetables, cereals and sugar plants; 0.02 mg/kg for nuts and oil plants; 0.05 mg/kg for herbs, tea, coffee, herbal infusions, spices and animal products [5]. In turn, carboxin (5,6-dihydro-2-methyl-1,4-oxathiin-3-carboxanilide) is the first synthetic fungicide applied to seeds, that allows to control of barley and wheat loose smut. It is an anilide produced by the condensation of the amino group of aniline with carboxylic groups of 2-methyl-5,6-dihydro-1,4-oxathiine-3-carboxylic acid. This pesticide is highly reactive and may be oxidized in the environmental conditions to carboxin sulfoxide and oxycarboxin. These metabolites have a very similar toxicity to the carboxin one [6]. The maximum allowed level of this pesticide, according to the EU regulations, is as follows: 0.03 mg/kg for fruits, animal products, vegetables, cereals and sugar plants; 0.05 mg/kg for nuts and oil plants; 0.1 mg/kg for herbs, tea, coffee, herbal infusions and spices [5]. 

There are various remediation methods for soils polluted with pesticides. They belong to various groups: (1) biological methods applying living organisms, (2) chemical methods (e.g., advanced oxidation process), (3) physical methods (e.g., adsorption), (4) mixed methods of the abovementioned techniques [4,7,8]. Biological methods include phytoremediation (using plants) and microbial remediation (using bacteria and microfungi). In both methods there are various species of organisms that may be used. They must be characterized by high colony growth rate, possess the ability to degrade or immobilize selected pesticides as well as be resistant to pesticides’ toxic activity. Among microorganisms used in bioremediation are: *Dechloromonas*, *Bacillus* and *Anthracophyllum* [7,9]. Chemical methods are based on the addition of various chemical compounds neutralizing a pesticide to soil. They result in transformation of pesticide or its derivatives to less harmful or nonharmful forms. One of the most common chemical methods used for contaminated soils is the advanced oxidation process (AOP), in which highly reactive radicals (e.g., OH·) are applied [4]. In physical methods various substances are used, but in contrast to chemical techniques, there is no pesticide transformation after their addition. Treated pesticide undergoes physical process: absorption (pesticide incorporation into added substance) and adsorption (pesticide adherence on the surface of applied solid). Adsorption is a very effective method of treating pesticide contamination. Based on specific interactions between adsorbent surface and adsorbed contaminants it is highly selective process, which does not require specialized apparatus [8,10]. Adsorption allows to bioavailability reduction of heavy metals and other hazardous compounds to soil organisms [11,12].

Nowadays, various types of adsorbents have been developed. They may be divided into specific groups: (1) natural materials (e.g., sawdust, wood); (2) natural materials modified to improve their textural parameters (e.g., activated biocarbons, silica gel); (3) manufactured materials (e.g., carbon-mineral composites, zeolites); (4) agricultural/industrial solid wastes (e.g., seeds, fruit peels, fly ash); and (5) biosorbents (e.g., chitosan) [8]. Among natural or treated materials, there are mixed adsorbents prepared using natural or treated clays/minerals (bentonite, kaolinite, diatomite, sepiolite, vermiculite etc.) and organic compounds [13]. Dolomite with addition of tea waste [14] and bentonite with addition of biochar [15] are examples of such materials. Development of mixed adsorbents is a very ecological trend. These solids are environmentally friendly, produced using various feedstock. Biochar is an example of organic compound, which can be mixed with various minerals. This carbonaceous material obtained from biomass is usually characterized by high adsorption capacity relative to inorganic and organic substances [16,17,18]. Mixed adsorbents may be used in wastewater treatment and soil remediation technologies [19,20]. Usually, they are obtained in two steps: mixing mineral with biomass and pyrolysis of prepared mixture at high temperature [15].

This paper focused on the adsorption mechanism of diuron and carboxin on the surface of biochar and goethite. The solids were selected for the study as potential compounds of mixed adsorbent. The authors prepared a simple mixture of these solids and performed adsorption experiments on the obtained material. The study included characterization of the solids (using SEM, FTIR, XPS, N_2_ adsorption/desorption, Boehm titration), adsorptive measurements (kinetics and isotherms) as well as adsorption data modeling. The adsorption was determined in the simple and mixed systems, i.e., containing one or two pesticides at the same time. In this way the adsorption affinity of diuron and carboxin to the selected solids was compared. The pesticides selected for this research are substances that can be leached from the soil into the groundwater. The addition of biochar, goethite or their mixture to the soil can significantly reduce this phenomenon and protect aquatic ecosystems from contamination. It is also worth mentioning that the biochar used in the experiments was obtained from agricultural waste—sunflower husk. In turn, the applied goethite is a by-product in hydrometallurgical processes, mainly zinc production. In this way the paper refers to the assumptions of the circular economy, which is based on minimizing the consumption of raw materials and the amount of waste, emission and loss of energy by creating a closed loop of processes. The waste from some processes should be used as raw materials for others, which minimizes the total waste production.

## 2. Materials and Methods

### 2.1. Pesticides

Diuron (CAS No. 330-54-1) and carboxin (CAS No. 5234-86-4), delivered by Sigma Aldrich Company (Saint Louis, MO, USA), were used in the study as adsorbates. The stock solutions, prepared using demineralized water, of diuron and carboxin were of concentrations equal to 10 and 100 mg/L, respectively. 

### 2.2. Adsorbent Preparation and Characteristics

Biochar (BC), goethite (G) and goethite–biochar mixture (G + BC) were used in the experiments as adsorbents. Biochar was prepared from sunflower husks by NEW TECHNOLOGY TRADE (Poland) by pyrolysis process of biomass at 650 °C. Goethite was delivered by Sigma-Aldrich Company (Saint Louis, MO, USA; CAS 20344-49-4). Before the experiments, biochar was crushed in a porcelain mortar (Jipo, Desná, Czech Republic) and sieved through 2 mm. Both biochar and goethite were washed to the conductivity below 2 µS/cm. The goethite–biochar mixture was prepared in three-step procedure. The first was the mechanical grinding of the mixture of goethite (85%) and biochar (15%) in a porcelain mortar, the second preparation of the mixture in 500 mL of demineralized water and 15 min sonication in ultrasonic bath (Emag, Mörfelden-Walldorf, Germany), and the third 3 h mixing of the prepared suspension (at 100 rpm speed) and drying at 105 °C.

Biochar, goethite and their mixture were characterized using several methods. Specific surface area (*S_BET_*) and porosity parameters (*V_t_*—total pore volume, *D*—average pore diameter) of the solids were determined using nitrogen adsorption/desorption method (ASAP 2420, Micromeritics Inc., Norcross, GA, USA). The *S_BET_* parameter was calculated using BET method, whereas textural parameters were calculated using desorption isotherms and BJH method. All probes were dried and out-gassed at 105 °C for 12 h before the measurement.

The amount of acidic and basic surface groups of adsorbents were examined by Boehm titration [21,22]. XPS (X-ray photoelectron spectroscopy) apparatus (Gammadata Scienta, Uppsala, Sweden) was used to determine elemental composition of biochar and goethite. The mixture could not be analyzed using XPS due to its high heterogeneity. The SEM images of adsorbents were made using scanning electron microscope PhenomX (Pik Instruments, Thermo Fisher Instruments, Somerset, NJ, USA). The surface groups of the solids were also determined using Nicolet 8700A FTIR (Fourier transform infrared spectroscopy) analyzer coupled with the Raman Nicolet NXR FT module (Thermo Scientific, Somerset, NJ, USA).

### 2.3. Adsorption Kinetics

The probes for kinetic measurements consisted of 0.05 g of the solid, 0.001 M of CaCl_2_ (supporting electrolyte) and 5 mg/L of selected pesticide (carboxin or diuron). After pH adjustment to 6 ± 0.1, the suspensions were shaken for the appropriate time: 10, 30, 60, 120, 180, 240, 300, 360, 480, 600, 720, 840 and 960 min. After that, the solids were separated from the systems using paper (389, Ahlstrom Munktell, Helsinki, Finland) and syringe (nylon, 0.45 µm, Thermo Fisher Scientific, Waltham, MA, USA) filters. The amount of diuron and carboxin in the obtained clear solutions was determined using high-pressure liquid chromatography (Ultimate 3000, Dionex, Sunnyvale, CA, USA) equipped with DAD detector (diode-array detection) and column Hypersil Green PAH (Thermo Fisher Scientific, Waltham, MA, USA).

Experimental data were modeled using SciDavis software as well as pseudo-first [23] (Equation (1)) and pseudo-second order [24] (Equation (2)) equations:

(1) $$\ln\left(q_{e}-q_{t}\right)=lnq_{e}-k_{1}\times t$$

(2) $$\frac{t}{q_{t}}=\frac{1}{k_{2}\times q_{e}^{2}}+\frac{t}{q_{e}}$$

where: *q_t_* (mg/g) is the pesticide adsorption capacity at time *t* (min); *q_e_* (mg/g) is the pesticide adsorption capacity at equilibrium; *k*_1_ (1/min) and *k*_2_ (g/mg·min) are the rate constants.

Adsorption kinetics of pesticides on the selected solids was also described using the intra-particle diffusion (IPD) model [25]:

(3) $$q_{t}=k_{D}\times t^{\frac{1}{2}}+C$$

where: *C* (mg/g) is a constant; *k_D_* (g/mg·min^1/2^) is the IPD rate constant. 

### 2.4. Adsorption Isotherms

The samples for measuring the adsorbed amount at equilibrium consisted of 0.05 g of the solid, 0.001 M of CaCl_2_ and pesticide with the concentration of 1, 2, 3, 5, 7 or 9 mg/L. The adsorption was conducted under shaking conditions for 24 h to reach an equilibrium state in all samples. Then, the suspensions were filtered and pesticide concentration in the solutions was determined using HPLC.

The obtained adsorption isotherm data were fitted to the 4 theoretical models, i.e., Langmuir (Equation (4)), Freundlich (Equation (5)), Langmuir–Freundlich (Equation (6)) and Redlich–Peterson (Equation (7)) using SciDavis software. The first one [26] is expressed as:

(4) $$q_{e}=\frac{Q_{m}K_{L}C_{e}}{1+K_{L}C_{e}}$$

where: *Q_m_* is the maximum amount of pesticide adsorbed in the monomolecular layer (mg/g); *K_L_* is the Langmuir constant (L/mg). 

To predict if the pesticide adsorption on the selected adsorbents is ‘favorable’ or ‘unfavorable’, a dimensionless separation factor (*K_R_*) was calculated using the formula [27]:

(5) $$K_{R}=\frac{1}{1+K_{L}C_{0}}$$

where: *C*_0_—the initial adsorbate concentration (mg/L). 

Based on the *K_R_* parameter, the shape of the isotherm can be determined accordingly: *K_R_* > 1—unfavorable isotherm, *K_R_* = 1—linear, 0 < *K_R_* < 1—favorable, *K_R_* = 0—irreversible.

The Freundlich isotherm [28] is expressed as:

(6) $$q_{e}=K_{F}\;{\left[\frac{C_{e}}{\;a_{m}}\right]}^{n}$$

where: *q_e_* is the amount of adsorbed pesticide at equilibrium (mg/g); *C_e_* is the equilibrium concentration of adsorbate in the solution (mg/L); *K_F_* (in units of *q_e_*) and *n* (0 *< n <* 1) are the Freundlich constants (mg/g(L/mg)^1/n^).

The Langmuir–Freundlich isotherm [27] is defined as:

(7) $$\frac{q_{e}}{A_{m}}={\left\{\frac{{\left(K_{LF}C_{e}\right)}^{m}}{\left[1+{\left(K_{LF}C_{e}\right)}^{m}\right]}\right\}}^{\frac{n}{m}}$$

where *K_LF_* is the constant of the Langmuir–Freundlich equation (L/mg); *A_m_* is the amount of available surface sites (mg/g); *n* and *m* (0 < *n*; *m* ≤ 1) are the parameters characterizing the shape of energy distribution function.

The linear form of Redlich–Peterson [29] isotherm model is defined as:

(8) $$\ln\left(K_{RP}\frac{C_{e}}{q_{e}}-1\right)=b_{RP}lnC_{e}+lna_{RP}\;$$

where: *K_RP_* is the Redlich–Peterson adsorption capacity constant; *a_RP_* is the Redlich–Peterson isotherm constant; *b_RP_* is the exponent between 0 and 1.

The efficiency (*E*, %) of pesticide adsorption was calculated using the equation [30]:

(9) $$E=\frac{C_{A}}{C_{0}}\times 100\%$$

where: *C_A_* is the concentration of pesticide adsorbed on the solid surface (mg/L); *C*_0_ is the initial pesticide concentration in the sample (mg/L).

### 2.5. Adsorption in the Mixed Systems

The composition of the samples was as follows: 0.05 g of the solid, 0.001 M CaCl2, 5 mg/L of carboxin and 5 mg/L of diuron. After pH adjustment to 6 and 24 h adsorption, the probes were filtered and analyzed using HPLC. The obtained results were not modeled due to high complexity of the examined systems. 

### 2.6. Statistical Analysis

All adsorptive measurements were made in triplicate. The measurement uncertainty was calculated using the Statistica software. 

## 3. Results and Discussion

### 3.1. Adsorbent Characterization

**Figure 1:**
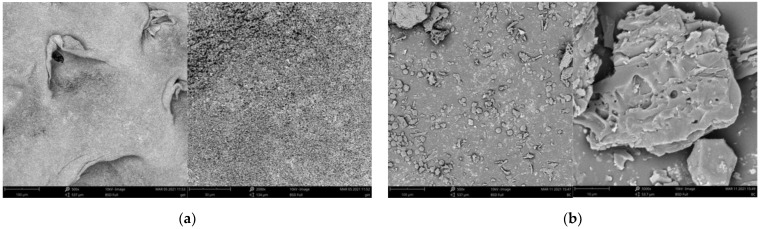

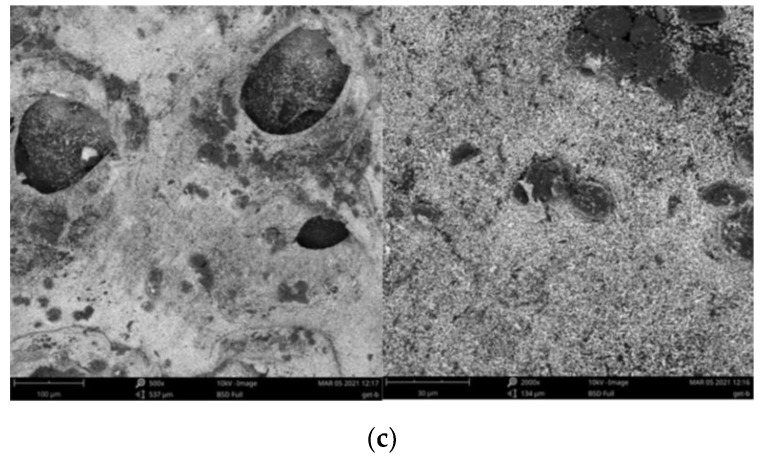
SEM images for goethite (**a**), biochar (**b**) and goethite–biochar mixture (**c**) made with magnification of 500, 2000 or 5000×.

The morphology of goethite, biochar and their mixture was observed using scanning electron microscope. The images of these materials are presented in Figure 1.

Porosity parameters, determined by N_2_ adsorption/desorption method, are summarized in Table 1. 

They showed that all adsorbents were characterized by poorly developed surface. The biochar had the largest pores (of average diameter close to small macropores—49.45 nm) among all tested solids. The pore volume and specific surface area were the lowest for this adsorbent (0.0025 cm^3^/g and 7.02 m^2^/g, respectively). On the other hand, the obtained goethite–biochar mixture was characterized by the smallest average pore diameter (9.51 nm) and the highest specific surface area (48.48 m^2^/g). 

Boehm titration allowed to determine the amount of acidic (lactonic, carboxylic and phenolic) and basic groups on the surface of selected solids. The obtained results are presented in Table 1. They indicated that basic moieties prevailed on the biochar surface. It contained 2.9 mmol/g of acidic groups and 3.2 mmol/g of basic ones. In turn, the mixture contained 0.4 mmol/g of acidic and 0.55 mmol/g of basic groups. Goethite is characterized by a specific structure, where oxygens are singly, doubly and triply coordinated, whereas all iron atoms are octahedrally coordinated. Thus, ≡Fe_2_OH groups predominate on the mineral surface [31]. 

**Figure 2:**
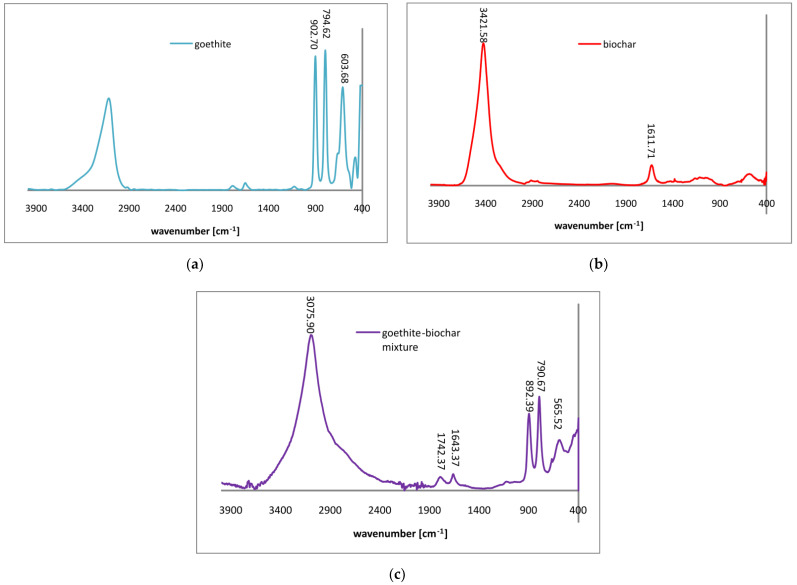
FTIR spectra for goethite (**a**), biochar (**b**) and goethite–biochar mixture (**c**).

The FTIR spectra obtained for goethite, biochar and their mixture are presented in Figure 2. 

The spectrum of goethite consisted of the following bands at: 3109.06 cm^−1^ (corresponding with stretching of OH groups arranged in hydrogen bond formation), 902.70 and 794.62 cm^−1^ (corresponding with FeOOH binding) and 603.68 cm^−1^ (corresponding with FeO stretching) [32]. The spectrum of biochar contained bands at: 3421.58 cm^−1^ (corresponding with stretching of OH groups forming hydrogen bonds) and 1611.71 cm^−1^ (which can be attributed to C=O vibrations in carboxylic and lactone groups as well as C=C stretching in aromatic rings). The bands at 1742.37 and 1643.37 cm^−1^ visible in the spectra of goethite–biochar spectrum corresponds with the same vibrations as the 1611.71 cm^−1^ one. 

**Figure 3:**
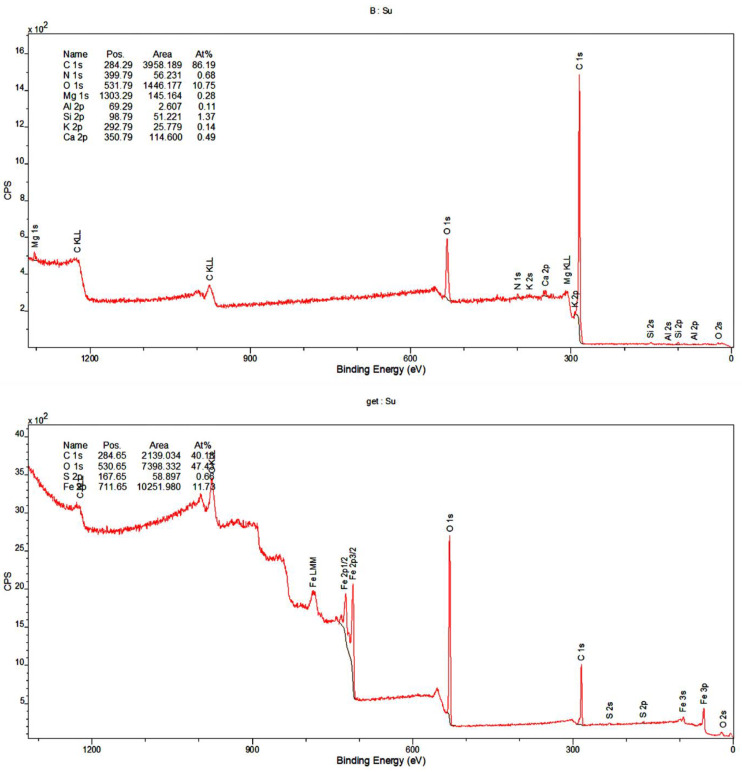
Survey XPS spectra for biochar (B) and goethite (get).

The same functional groups were determined using XPS apparatus. Thus, the results of FTIR and XPS were consistent. Additionally, the obtained XPS data confirmed the presence of mineral compounds in the biochar sample. There were the following species: Al–O (alumina), Al–OH (aluminum hydroxyl groups), Si–O–Si (silica/silicates), Mg–O (magnesium-oxygen bond) and Si–OH (silica hydroxyl groups). The survey XPS spectra as well as O1s XPS data for biochar and goethite are presented in Figure 3 and Table 2. The peaks were described based on the literature [33,34,35,36,37,38,39].

### 3.2. Adsorption Mechanism of Carboxin and Diuron on the Goethite and Biochar Surface

**Figure 4:**
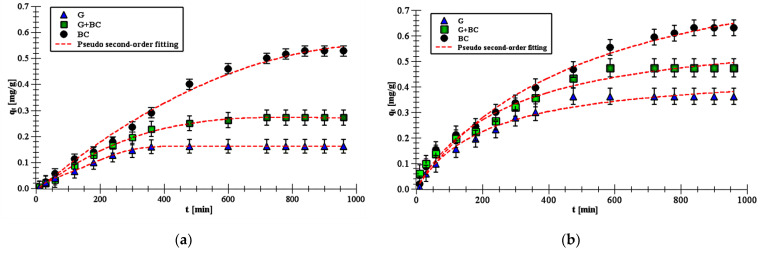
Adsorption kinetics data of diuron (**a**) and carboxin (**b**) on the goethite, biochar and goethite–biochar mixture (points) as well as pseudo second-order fitting (lines) at pH 6.

The experimental curves of pesticide adsorption kinetics with the fitting to pseudo-second order equation are presented in Figure 4. They showed that the 24 h were sufficient to reach equilibrium. When the plateau was reached, there was no change in the adsorption amount over time. So, the adsorbent could not adsorb more adsorbate molecules. 

All kinetics adsorption parameters, correlation coefficients and errors are presented in Table 3 and showed clear differences among applied adsorbents. 

Experimental kinetics data were fitted to three theoretical kinetic equations: the pseudo first-order (Equation (1)); the pseudo second-order (Equation (2)); and the intra-particle diffusion (IPD) model (Equation (3)). The best fitting was obtained to the pseudo second-order model (R^2^ > 0.99). This suggests chemisorption process of carboxin and diuron on goethite, biochar and goethite–biochar mixture. What is more, this indicated that the adsorbent surfaces were heterogeneous [24]. 

The carboxin and diuron adsorption process was the fastest on goethite, whereas it was the slowest on biochar. The k_2_ values of carboxin adsorption decreased in the range 0.85–0.44·10^−2^ g/mg·min and the k_2_ values of diuron adsorption decreased in the range 0.79–0.19·10^−2^ g/mg·min. This difference between adsorbents was probably dictated by various number of their active sites. Goethite is characterized by smaller number of active sites and, as a consequence, the equilibrium of adsorption can be achieved faster than in the system containing biochar. Additionally, the mineral adsorbed smaller amounts of carboxin and diuron than organic solid. The *q_e_* value on biochar was 0.64 mg/g for carboxin and 0.53 mg/g for diuron. In turn, the *q_e_* value on goethite was 0.37 and 0.16 mg/g for carboxin and diuron, respectively. This parameter increased after biochar addition and equaled 0.49 mg/g for carboxin and 0.27 mg/g for diuron for the goethite–biochar mixture. The above results also suggested that carboxin adsorbed on the solids in higher amounts than diuron.

**Figure 5:**
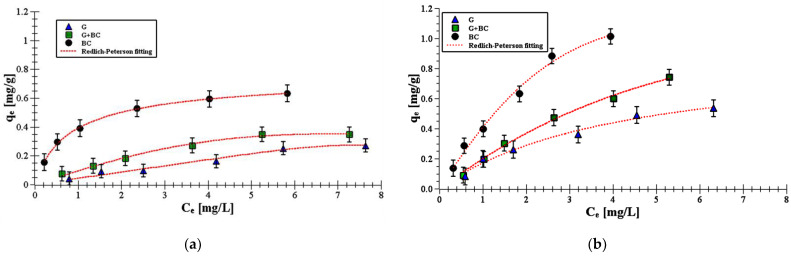
Adsorption equilibrium data of diuron (**a**) and carboxin (**b**) on the goethite, biochar and goethite–biochar mixture (points) as well as Redlich–Peterson fitting (lines) at pH 6.

Figure 5 presents experimental isotherms with the fits obtained from Redlich–Peterson model.

The equilibrium adsorption data of carboxin and diuron adsorption process on goethite, biochar and their mixture were analyzed using four models: the Freundlich (Equation (6)), Langmuir (Equation (4)), Langmuir–Freundlich (Equation (7)) and Redlich–Peterson (Equation (8)) ones. The obtained adsorption isotherm parameters were summarized in Table 4.

The Redlich–Peterson isotherm model yielded R^2^ > 0.99 for all systems. This indicated that the model can be used to describe experimental adsorption data. The Redlich–Peterson is a three-parameter isotherm connecting Langmuir and Freundlich isotherms assumptions. A good fitting to this model means that the adsorption mechanism is a mix and does not follow ideal monolayer adsorption [40]. If the values of b_RP_ will be close to 1, then the Langmuir will be fitted, whereas *b_RP_* will be close 0, the Freundlich isotherm will be suitable [41]. The exponent *b_RP_* for carboxin and diuron adsorption vary between 0.22–0.08. So, there is a predominance of multilayer adsorption. 

**Figure 6:**
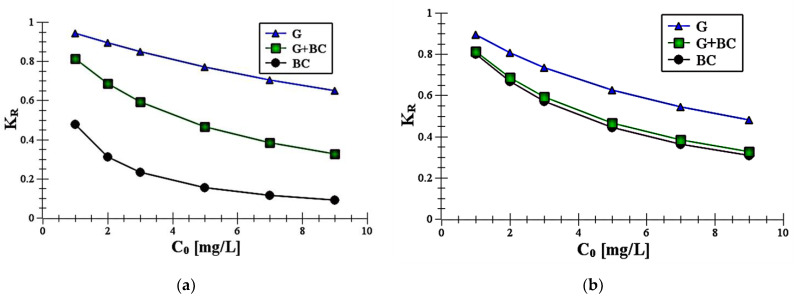
Plot of K_R_ parameter against initial diuron (**a**) and carboxin (**b**) concentration *C*_0_.

The separation factors *K_R_* was calculated by Equation (5). The values, presented in Figure 6, were <1 for all adsorbents, which showed that the carboxin and diuron adsorption by the all adsorbents was favorable. 

The obtained data suggested that adsorption process of carboxin and diuron was the most favorable on biochar. After addition of biochar to goethite the *K_R_* values increased, but they were lower than the values noted for goethite. This reflected that adsorption of carboxin and diuron was more favorable on the mixture than on the mineral. In addition, the parameters observed for diuron on biochar were lower than those for carboxin. It suggested that diuron adsorption was more favorable on the experimental organic surface than carboxin one. 

The obtained results proved that selected pesticides were adsorbed on carbonaceous material, mineral as well as the mixture of carbonaceous material and mineral. Probably, the mechanism of carboxin/diuron adsorption is based on the creation of hydrogen bonds and donor–acceptor interactions between substituents on the solid surface and substituents of pesticide molecules [42]. Carboxin and diuron adsorbed better on biochar and the goethite–biochar mixture because of increased hydrophobic nature and presence of aromatic rings substituted with various groups within the solid particles. Donor-acceptor interaction was formed between amino groups of pesticides and hydroxyl groups of solid surface or between carbonyl groups of pesticides and adsorbent hydroxyl groups. The π–π electron-donor–acceptor interactions may also occur between aromatic rings of pesticides and biochar [42]. As it was mentioned above, in the single solution, carboxin was adsorbed in higher amounts than diuron. It was caused by larger number of hydrogen bond acceptors in the case of this pesticide. This parameter equaled 3 for carboxin and 1 for diuron. The number of hydrogen donors was the same for both pesticides and equal to 1 [43].

### 3.3. Carboxin and Diuron Adsorption on the Goethite and Biochar Surface in the Mixed Pesticide Solution

**Figure 7:**
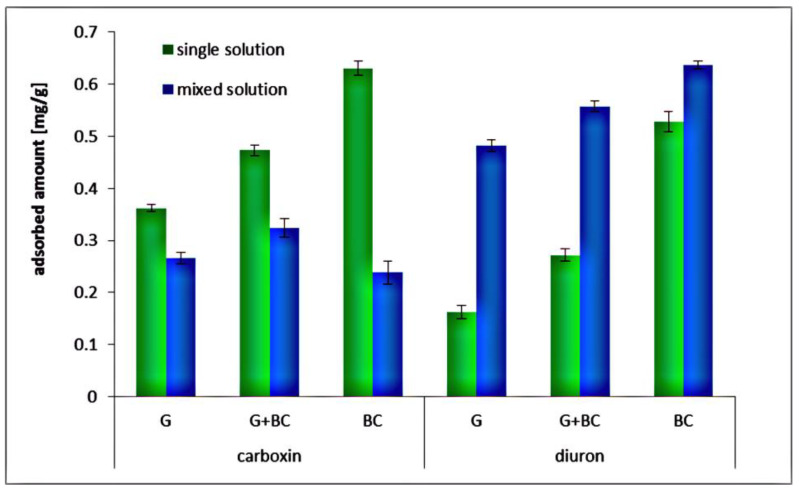
Adsorbed amount of diuron and carboxin on goethite, biochar and goethite–biochar mixture in the mixed pesticide solutions, at pH 5.

The adsorbed amounts of carboxin and diuron noted in the mixed solutions, i.e., containing both pesticides simultaneously, are presented in Figure 7. 

They showed that diuron was adsorbed in higher amounts in the mixed solution than in the single ones. On the other hand, the carboxin showed a different tendency—it was adsorbed in larger amounts in the single systems than in the mixed ones. The observed differences were dictated by various specific area of pesticide molecules. It equals 63.6 Å for carboxin and 32.3 Å for diuron [43]. This means that diuron molecules are smaller and can be bounded to the surface faster. As a result, diuron molecules take up active sites and make them inaccessible to carboxin. Increased adsorption level of diuron in the mixed systems (compared to those noted in the solutions containing only one adsorbate) is probably dictated by formation of second and further pesticide layers on the solid surface. Within this structure one carboxin molecules may accelerate diuron adsorption by π–π electron-donor-acceptor interactions.

## 4. Conclusions

The performed study allowed to formulate the conclusions:

1. The pseudo second-order model best fitted experimental data (*R^2^* > 0.99). This suggests chemisorption process of carboxin and diuron on goethite, biochar and goethite–biochar mixture.
2. The carboxin and diuron adsorption process was the fastest on goethite, whereas it was the slowest on biochar. This is probably related to the number of active sites on the adsorbents.
3. Carboxin adsorbed on the solids in higher amounts than diuron. The *q_e_* value on biochar was 0.64 mg/g for carboxin and 0.53 mg/g for diuron. In turn, the *q_e_* value on goethite was 0.37 and 0.16 mg/g for carboxin and diuron, respectively.
4. The Redlich–Peterson isotherm model best described experimental data of carboxin/diuron adsorption on biochar, goethite and goethite–biochar mixture.
5. The obtained K_R_ parameters suggested that adsorption process of carboxin and diuron was the most favorable on biochar.
6. Carboxin/diuron adsorption is based on the creation of hydrogen bonds and donor-acceptor interactions between substituents on the solid surface and substituents of pesticide molecules. The π–π electron-donor–acceptor interactions may also occur between aromatic rings of pesticides and biochar.
7. In the mixed solution, diuron was adsorbed on the selected solids in higher amounts, whereas carboxin in lower amounts than in the single systems. This is probably associated with different molecule area of used pesticides as well as formation of adsorption multilayer, within carboxin accelerates diuron bonding.
8. Goethite and sunflower husks, as waste from the metallurgical industry and agriculture, respectively, can be used to prepare environmentally friendly adsorbents, capable of binding carboxin and diuron.

## Figures and Tables

**Table 1:** Specific surface area, porosity parameters and acidic/basic group content for adsorbents.

Parameter	Goethite	Biochar	Goethite–Biochar Mixture
*S_BET_* (m^2^/g) ^1^	11.10	7.02	48.48
*V_t_* (cm^3^/g) ^1^	0.028	0.0024	0.029
*D* (nm) ^1^	12.14	49.45	9.51
Acidic group content ^2^ (mmol/g)	-	2.9	0.4
Basic group content ^2^ (mmol/g)	-	3.2	0.55

^1^ determined using nitrogen adsorption/desorption method, ^2^ determined using Boehm titration.

**Table 2:** O1s XPS data obtained for goethite and biochar.

Material	Position	Concentration (%)	Species	Description
Goethite	530.44	40.9	Fe–O–Fe	Iron oxide
	531.79	54.8	Fe–OH	Iron hydroxide
	533.6	4.3	H_2_O	Water
Biochar	528.1	6.5	O^−2^	Oxide anion/metal oxides
	529.6	12.0	quin O^−2^ Me–O–C	Quinones Oxide anion/metal oxides Carbonates
	531.7	43.8	O=C–O^− ^ O=C	Carboxyl groups Carboxyl groups
	533.4	36.3	Al–O Al–OH Si–O–Si Mg–O	Alumina Aluminum hydroxyl groups Silica/silicates Magnesium-oxygen bond
	534.7	1.4	Si–OH C–OH O=C–O^−^ H_2_O/O_2_	Silica hydroxyl groups Hydroxyl groups (aromatic) Carboxyl groups Water/adsorbed oxygen

**Table 3:** Kinetic parameters of pesticides adsorption on goethite (G), biochar (BC) and goethite–biochar mixture (G + BC).

Kinetic Equation	Parameter	Carboxin			Diuron		
		G	G + BC	BC	G	G + BC	BC
Pseudo-first order	k_1_ × 10^−2^ (1/min)	0.48 ± 0.07	0.34 ± 0.04	0.25 ± 0.01	0.75 ± 0.01	0.49 ± 0.04	0.21 ± 0.05
	*q_e_* (mg/g)	0.37 ± 0.07	0.43 ± 0.21	0.59 ± 0.21	0.19 ± 0.07	0.31 ± 0.01	0.54 ± 0.01
	*R* ^2^	0.990	0.982	0.981	0.863	0.971	0.987
Pseudo-second order	k_2_ × 10^−2^ (g/mg·min)	0.85 ± 0.07	0.69 ± 0.07	0.44 ± 0.03	0.79 ± 0.07	0.36 ± 0.07	0.19 ± 0.03
	*q_e_* (mg/g)	0.37 ± 0.04	0.49 ± 0.03	0.64 ± 0.04	0.16 ± 0.04	0.27 ± 0.03	0.53 ± 0.04
	*R^2^*	0.998	0.998	0.996	0.998	0.996	0.999
Intra-particle diffusion model	*k_D_* × 10^−2^ (g/mg·min^1/2^)	2.31 ± 0.04	1.63 ± 0.06	1.29 ± 0.12	1.92 ± 0.04	0.88 ± 0.06	0.58 ± 0.09
	*R^2^*	0.921	0.963	0.925	0.847	0.885	0.922

**Table 4:** Isotherm parameters of pesticides adsorption on the goethite (G), biochar (B) and goethite–biochar mixture (G+BC).

Isotherms	Parameter	Carboxin			Diuron		
		G	G + BC	BC	G	G + BC	BC
Freundlich	*K_F_* (mg/g(L/mg)^1/n^)	0.18 ± 0.02	0.21 ± 0.02	0.41 ± 0.03	0.05 ± 0.01	0.12 ± 0.02	0.36 ± 0.02
	1/*n*	0.78 ± 0.18	0.69 ± 0.09	0.62 ± 0.14	0.81 ± 0.16	0.59 ± 0.24	0.35 ± 0.05
	*R^2^*	0.971	0.988	0.979	0.962	0.953	0.959
Langmuir	*K_L_* (L/mg)	0.12 ± 0.06	0.23 ± 0.03	0.25 ± 0.06	0.06 ± 0.04	0.23 ± 0.06	1.10 ± 0.02
	*Q_m_* (mg/g)	0.91 ± 0.12	1.93 ± 0.29	2.08 ± 0.31	0.59 ± 0.13	0.73 ± 0.07	0.94 ± 0.01
	*R^2^*	0.982	0.994	0.988	0.962	0.979	0.999
Langmuir–Freundlich	*K_LF _* (L/mg)	0.32 ± 0.15	0.49 ± 0.11	0.65 ± 0.06	0.03 ± 0.01	0.24 ± 0.08	0.25 ± 0.07
	*A_m_* (mg/g)	0.49 ± 0.05	0.68 ± 0.07	1.77 ± 0.27	0.26 ± 0.12	0.55 ± 0.13	1.83 ± 0.44
	*m*	1.39 ± 0.58	1.29 ± 0.55	1.06 ± 0.05	1.02 ± 0.52	0.89 ± 0.10	0.52 ± 0.08
	*R^2^*	0.983	0.997	0.989	0.963	0.979	0.998
Redlich–Peterson	*K_RP _* (L/g)	0.95 ± 0.22	3.75 ± 0.62	5.61 ± 0.72	0.65 ± 0.31	2.09 ± 0.76	6.39 ± 0.72
	*a_RP _* (L/mg)	1.02 ± 0.17	1.33 ± 0.44	1.59 ± 0.51	1.08 ± 0.12	1.31 ± 0.27	1.53 ± 0.23
	*b_RP_*	0.22 ± 0.03	0.16 ± 0.01	0.08 ± 0.01	0.20 ± 0.01	0.12 ± 0.01	0.09 ± 0.03
	*R^2^*	0.992	0.998	0.991	0.990	0.991	0.991

## Data Availability

The reported results can be found at the Institute of Agrophysics, PAS, Lublin, Poland.
